# 92. Clinical Decision-Making about Chronic Antibiotic Suppression after Prosthetic Joint Infection Treatment: Qualitative Insights for Antibiotic Stewardship

**DOI:** 10.1093/ofid/ofab466.294

**Published:** 2021-12-04

**Authors:** Kimberly Dukes, Julia Walhof, Poorani Sekar, Rajeshwari Nair, Hiroyuki Suzuki, Daniel Suh, Stacey Hockett Sherlock, Bruce Alexander, Kelly Richardson, Brice Beck, Heather Schacht Reisinger, Andrew Pugely, Mireia Puig-Asensio, Marin Schweizer

**Affiliations:** 1 Iowa City VA, Iowa City, Iowa; 2 Iowa City VA Health Care System, Iowa City, Iowa; 3 University of Iowa Carver College of Medicine, Iowa City, Iowa; 4 The University of Iowa Carver College of Medicine, Iowa City, Iowa; 5 University of Iowa Hospitals and Clinics, Iowa City, Iowa; 6 VA Iowa City Health Care System and University of Iowa, Iowa City, Iowa; 7 University of Iowa, Iowa City, Iowa; 8 University of Iowa Hospital and Clinics, Iowa City, Iowa; 9 University of Iowa Hospitals & Clinics, Iowa City, IA

## Abstract

**Background:**

Patients who develop prosthetic joint infections (PJI) may be prescribed chronic antibiotic suppression (CAS) ( > 6 months of antibiotics) after initial antibiotic treatment for the PJI. Patients at low risk of recurrent infection may be good targets for antibiotic stewardship. De-implementation of CAS could potentially reduce the emergence of antibiotic resistant organisms and decrease antibiotic-associated adverse events. Our ongoing study aims to characterize clinical decision-making processes about CAS prescribing and identify points for antibiotic stewardship interventions to stop CAS prescribing for patients who will not benefit.

**Methods:**

We conducted semi-structured interviews with 33 physicians and nurses at 8 Veterans Affairs hospitals, chosen for variation in hospital size, complexity, region, and CAS prescribing. Interviewees included orthopedic surgeons, infectious disease (ID) physicians, hospital epidemiologists, nurses, nurse managers, and primary care physicians (PCPs). We conducted inductive, consensus-based thematic analysis on interview transcripts, using the program MAXQDA.

**Results:**

Participants reported a complex decision-making process that included a range of collaborative approaches with other clinicians and patients. Their risk-benefit calculation for CAS usually included the type of revision surgery performed, the evidence base, the organism, and patient factors. Surgeons and ID physicians, the primary CAS prescribers, collaborated variably and sometimes consulted pharmacists or antibiotic stewards. Participants emphasized the importance of clinician autonomy and buy-in to order to effect practice change based on evidence, rather than top-down policies. They identified other significant time points that occurred before or after the CAS prescribing decision (initial PJI treatment decisions, follow-up appointments) and identified other decision makers about CAS maintenance (e.g., patients, PCPs). (See Figure 1).

Figure 1. Decision Points Relevant to Prescribing or Maintenance of Chronic Antibiotic Suppression after PJI. PJI, prosthetic joint infection; ID, Infectious Diseases physician; PCP, primary care physician; IV, intravenous

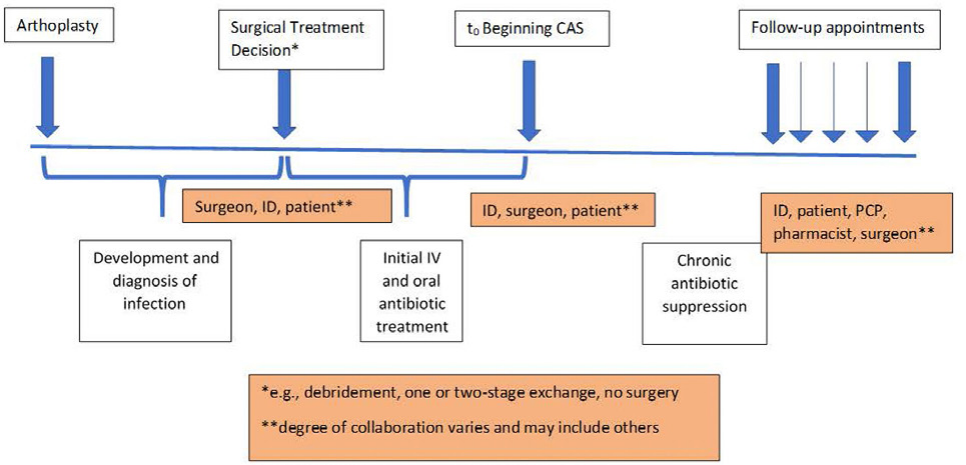

**Conclusion:**

Interventions to optimize CAS prescribing should incorporate clinician concerns about prescriber autonomy and a preference for collaborative decision-making as well as understanding the range of decision makers across time.

**Disclosures:**

**Daniel Suh, MS MPH**, **General Electric** (Shareholder)**Merck** (Shareholder)**Moderna** (Shareholder)**Smile Direct Club** (Shareholder) **Bruce Alexander, PharmD**, **Bruce Alexander Consulting** (Independent Contractor) **Andrew Pugely, MD, MBA**, **Globus Medical** (Research Grant or Support)**Medtronic** (Consultant)**United Healthcare** (Consultant) **Marin Schweizer, PhD**, **3M** (Grant/Research Support)**PDI** (Grant/Research Support)

